# X-ray characterization of physical-vapor-transport-grown bulk AlN single crystals

**DOI:** 10.1107/S1600576720008961

**Published:** 2020-07-30

**Authors:** Thomas Wicht, Stephan Müller, Roland Weingärtner, Boris Epelbaum, Sven Besendörfer, Ulrich Bläß, Matthias Weisser, Tobias Unruh, Elke Meissner

**Affiliations:** a Fraunhofer IISB, Schottkystrasse 10, Erlangen, 91058, Germany; b Institute for Crystallography and Structural Physics, Friedrich-Alexander-Universität Erlangen-Nürnberg, Staudtstrasse 3, Erlangen, 91058, Germany; c Chair of Electron Devices, Friedrich-Alexander-Universität Erlangen-Nürnberg, Cauerstrasse 6, Erlangen, 91058, Germany

**Keywords:** aluminium nitride, X-ray topography, X-ray diffraction, dislocations

## Abstract

AlN slices from bulk crystals grown under low thermomechanical stress conditions via the physical vapor transport (PVT) method were analyzed by X-ray methods to study the influence of the growth mode on the crystal quality. Defect types and densities were analyzed along axial [0001] as well as lateral growth directions. X-ray diffraction (0110) rocking-curve mappings of representative wafer cuts reveal a low mean FWHM of 13.4 arcsec, indicating the generally high crystal quality. The total dislocation density of 2 × 10^3^ cm^−2^ as determined by X-ray topography is low and dislocations are largely threading edge dislocations of **b** = 1/3〈1120〉 type. The absence of basal plane dislocations in homogeneous crystal regions void of macroscopic defects can be linked to the low-stress growth conditions. Under the investigated growth conditions this high crystal quality can be maintained both along the axial [0001] direction and within lateral growth directions. Exceptions to this are some locally confined, misoriented grains and defect clusters, most of which are directly inherited from the seed or are formed due to the employed seed fixation technique on the outer periphery of the crystals. Seed-shaping experiments indicate no apparent kinetic limitations for an enhanced lateral expansion rate and the resulting crystal quality, specifically with regard to the growth mode on *a*-face facets.

## Abstract 2

AlN single crystals grown by physical vapor transport have been analyzed by X-ray methods to evaluate dislocation types, densities and spatial distribution within the crystal. Potential changes of the AlN crystal quality during growth, both within the axial growth direction and within lateral expansion areas, have been investigated.

## 1. Introduction   

There is increasing demand for single-crystal AlN wafers due to their potential as substrates for the epitaxy of AlGaN materials for UV-optoelectronics and power electronics. For the implementation of a high-yielding cost-effective manufacturing process for these AlGaN-based devices the availability of large high-quality AlN substrates is critical. Strong indications exist that AlN crystal growth on native AlN seeds via physical vapor transport (PVT) is the method of choice to address these requirements. Significant progress has been made quite recently with respect to the industrial production of low-defect-density PVT AlN substrates with a diameter of up to 2 inches (1 inch = 25.4 mm) and free of macroscopic defects (Dalmau *et al.*, 2019 ▸; Schowalter *et al.*, 2020 ▸; Bondokov *et al.*, 2019 ▸).

Despite these achievements, high-quality 2 inch AlN substrates are still far from being commercially widely available. Moreover, for applications in power electronics, substrate sizes of at least 100 mm will be necessary to take advantage of the economy of scale and the advanced equipment of existing fabrication lines. While a slow but steady increase of wafer diameters can be achieved by repeated PVT bulk growth (Hartmann, 2013 ▸; Raghothamachar *et al.*, 2012 ▸), limited data are available about the development of crystallographic defects along the axial ([0001] or [0001]) and lateral growth directions during diameter enlargement and the underlying mechanisms for defect formation. It has been pointed out (Hartmann *et al.*, 2020 ▸) that a careful design of axial and radial temperature gradients in the growth zone is necessary to reduce the density of grown-in dislocations. As a consequence, typically employed expansion angles (Dalmau *et al.*, 2018 ▸) allow only for a moderate diameter expansion within a single growth run to avoid degradation of the crystal quality. Within this context it is of scientific interest to separate the effects of thermal gradients or general thermomechanical stress from any potential kinetic limitations for the growth along relevant crystallographic directions of AlN.

As a proof of concept, we employed for this study a PVT growth geometry with low axial and radial thermal gradients and low absolute source-to-seed temperature differences as indicated by numerical heat-transfer simulations, and only local seed fixation at the outer periphery to minimize the effect of thermomechanical stress on the AlN crystal quality, also enabling growth towards the near-equilibrium shape. In this study, we have analyzed PVT-grown AlN samples by high-energy X-ray diffraction and X-ray topography. These complementary methods provide information about the real structure of the crystals, in particular the dislocation types, densities and spatial distribution within the crystal.

## 2. Experimental   

**Figure 1:**
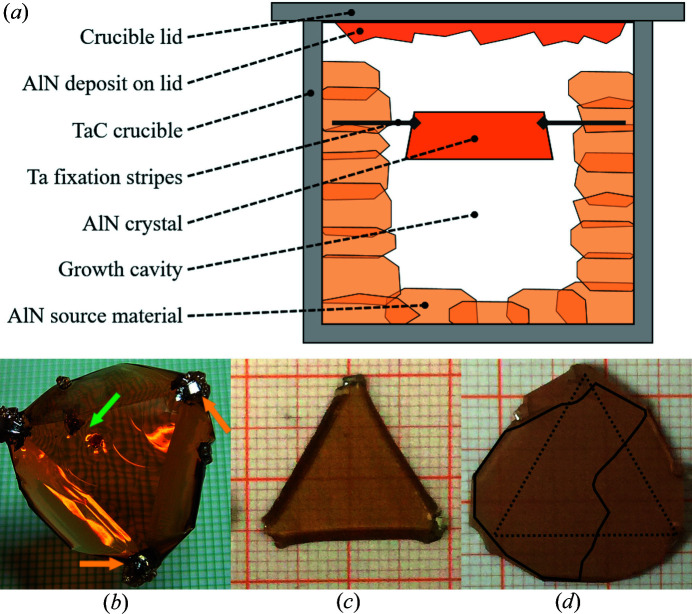
(*a*) Lely-type growth setup used at Fraunhofer IISB for the current study, (*b*) as-grown crystal boule B, (*c*) triangular seed B1 used to grow boule B and (*d*) wafer B4 cut from boule B. The dotted triangle in (*d*) illustrates the border between material grown axially on top of the seed and the lateral expansion area. The solid line encloses the fractured piece of B4 after surface preparation steps used for characterization.

Two characteristic AlN single crystals, fabricated at Fraunhofer IISB using PVT growth, were investigated. Both crystals (boules A and B) were grown on AlN seeds prepared from previously grown crystals under the same conditions. AlN PVT growth was performed in a Lely-type assembly (Lely, 1955 ▸), shown schematically in Fig. 1 ▸(*a*), with the specific goal of minimizing thermal gradients and vapor supersaturation near the growth interface. The main structure used for growth purposes was a central cavity surrounded by sintered blocks of coarse-grained (1 to 3 mm) AlN of high purity, inside a loosely closed TaC crucible. Due to the presence of TaC material the growth environment is considered as slightly C contaminated. The AlN charge material was carefully purified by repeated re-sublimation in W crucibles in a carbon-free reactor. Growth experiments were performed with a run time (*t*) between 24 and 48 h and 700–900 mbar (1 mbar = 100 Pa) of nitro­gen pressure in a furnace with indirect inductive heating of the crucible via a graphite susceptor. Temperatures (*T*) were measured by bottom and top optical pyrometry, with temperatures at the top of the crucible ranging from 2523 to 2623 K. The temperatures inside the crucible are not directly accessible, but numerical global heat-transfer simulations, verified by dedicated test configurations, indicated 50–60 K higher AlN seed temperatures and low *T* gradients close to the seed. Taking typical uncertainties for simulation results into account, we can estimate upper limits for the axial and radial *T* gradients near the growing crystal to be <5 K cm^−1^ and <1 K cm^−1^, respectively. On the basis of these conditions and additionally considering the small distances between the AlN source and the growth interface with corresponding absolute temperature differences of <3 K, the habit and faceting of grown crystals reflect differences in the growth rate in different crystallographic directions (considered as the Wulff shape). The largest portion of new material grew in the [0001] direction on the N-polar face (facing towards the hotter crucible bottom), while only 200–300 µm grew on the Al-polar side.

Crystal ‘A’ used in our study was grown on a self-produced rounded AlN seed wafer with a diameter of approximately 20 mm and grew into a hexagonal prism with approximately 25 mm diameter and 5 mm thickness. The seed for crystal ‘B’, shown in Fig. 1 ▸(*c*), was intentionally cut from a round seed into a nearly equilateral triangle shape with the sides parallel to three of the 〈1100〉 directions. This triggered an enhanced lateral growth, especially 〈1120〉 *a*-face growth, leading to a relatively fast transformation of the triangle seed towards a ‘standard’ hexagonal crystal shape. We point out that, in contrast to other AlN PVT growth results reported in the literature (Hartmann *et al.*, 2016 ▸; Dalmau *et al.*, 2011 ▸), the lateral expansion areas of the crystal in Fig. 1 ▸(*b*) show little to no difference in coloration in visible light compared with the central region. This is most likely connected to the significant differences in growth conditions, potentially affecting the incorporation of point defects. Further investigations of this phenomenon are worthwhile but are beyond the scope of this paper. Generally, all crystals produced with the described seed fixation typically suffer from polycrystalline inclusions near the edges, initiated by the fixation stripes [see yellow arrows in Fig. 1 ▸(*b*)].

Additionally, small parasitic crystals may appear within the main crystal body [green arrow in Fig. 1 ▸(*b*)]. These defective areas, corresponding to parasitic growth, are not the focus of our considerations within the more detailed analysis of the defect and dislocation structures in main areas of the grown crystal body. Actually, these small parasitic polycrystalline areas did not visibly disturb the main crystal volume during the growth process. However, upon cooling of the growth assembly to room temperature, they may produce local stress in the crystals grown relatively free of thermal stress, and may even trigger crystal or wafer cracking in subsequent processing steps. In particular, crystal B was harvested intact, but wafer B4, as shown in Fig. 1 ▸(*d*), broke in the course of wafering and polishing. Nevertheless, a large enough piece could be salvaged for further defect characterization (the shape of the investigated part of B4 is outlined).

After growth, both investigated boules A and B were cut into wafers of ∼600 µm thickness, for further surface preparation steps. The wafers are named in ascending order based on their distance to the seed, with A1 being the recovered original seed of crystal A.

To study the influence of increasing distance from the original seed on growth direction, wafers A3 and A4 of boule A were analyzed in more detail and compared with regard to their growth evolution. From boule B the wafer B4 was analyzed. As it exhibits high amounts of lateral growth, this allowed for a detailed investigation of the impact of the lateral growth mode on the material quality, in particular in comparison with areas of the same boule grown in the [0001] direction.

**Figure 2:**
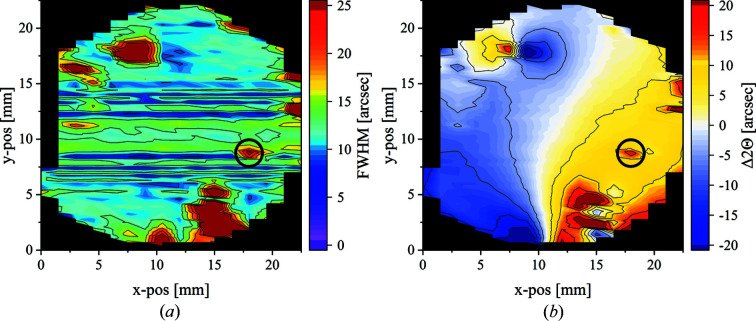
X-ray diffraction 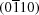 rocking-curve mappings of wafer A3. (*a*) FWHM mapping (values of 25 arcsec and above are indicated by a dark-red color). (*b*) Δ2Θ mapping (values less than −20 arcsec are indicated by a dark-blue color, values higher than 20 arcsec are indicated by a dark-red color). Visible horizontal lines seem to be artifacts related to data-smoothing procedures to create the color contour maps.

The wafers were analyzed by different X-ray diffraction experiments and defect-selective KOH–NaOH etching. X-ray measurements were carried out via a laboratory X-ray topography instrument and with a unique high-energy focusing Laue method at HEXbay laboratory at the Institute for Crystallography and Structural Physics in Erlangen. A detailed description of that measurement setup and principle can be found in the work of Stockmeier & Magerl (2008 ▸) and Weisser (2004 ▸). (0110) transmission Laue mappings were performed for wafer A3. From these, local FWHM and 2Θ-shift heat maps with ∼900 data points (each representing an area of 1.5 × 0.4 mm of the wafer) were extracted (Fig. 2 ▸). The final FWHM values were adjusted for both the instrument response function and broadening effects due to the sample thickness (both measured with a high-quality Si wafer).

Additional X-ray topography (XRT) measurements were performed with a Rigaku XRTmicron laboratory device. As a source, Cu and Mo rotating anodes were used at a power of 1.2 kW for measuring in reflection and transmission geometry, respectively. A 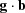 analysis of the visible contrast under different diffraction conditions was performed. As there are some geometric restrictions imposed by the equipment setup, the diffraction vectors chosen were 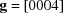 (reflection), 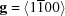 (transmission) and 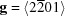 (transmission).

Defect-selective etching on the Al-polar side was performed on wafers A3 and A4 using a KOH–NaOH eutectic melt at a temperature of 723 K and with an etch time of 45 s. Optical microscopy as well as scanning electron microscopy (SEM) were used for analyzing the etched wafer surface.

## 3. Results and discussion   

Fig. 2 ▸(*a*) shows the FWHM mapping determined from the (0110) rocking curves measured for wafer A3. The color-scale range from purple to red illustrates FWHM values from 0 to 25 arcsec. High values of 25 arcsec and above are represented by a dark-red color.

**Figure 3:**
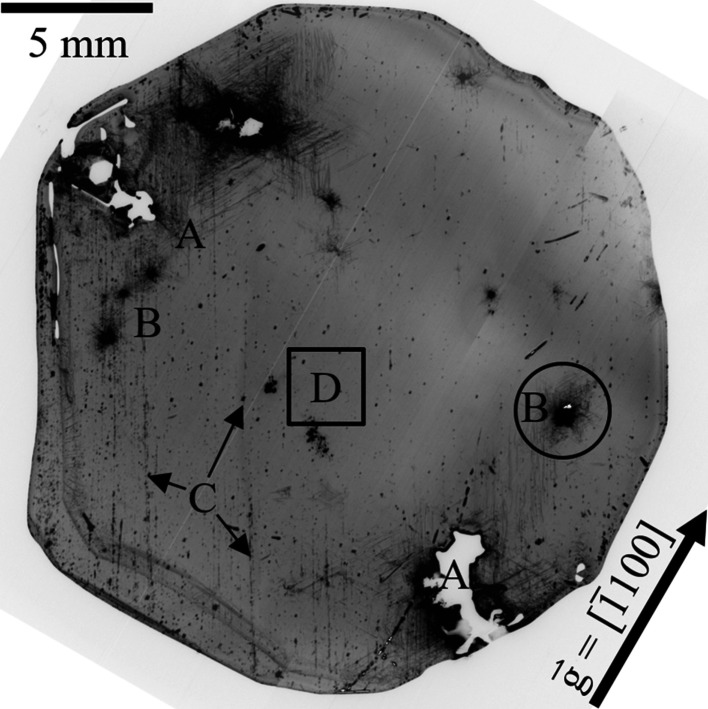
XRT image of A3 with 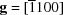 in transmission mode. A: Misoriented grains; B: defect cluster; C: residual surface damage from surface preparation steps; D: area void of macroscopic defects used for the determination of the dislocation density (Fig. 4 ▸).

FWHM values of over 80% of the wafer area did not exceed values of 15 arcsec. The mean FWHM is as low as 13.4 arcsec. These results indicate that the tilt of the prism planes (rotation around the [2110] axis) seems to be rather low. This agrees with the XRT results, indicating that basal plane dislocations (BPDs) seem to be absent for most of the wafer volume (Fig. 3 ▸). In general there is no significant change in the FWHM values from the center to the edge of the wafer.

High FWHM values exceeding 25 arcsec are almost exclusively present in macroscopic misoriented regions (*e.g.* bottom right of Fig. 3 ▸). One exception to this observation seems to be the area marked with the black circle, which exhibits values of up to 27 arcsec. However, no abnormalities could be observed in this region via optical microscopy. Additional XRT measurements were conducted in this region to check for the presence of dislocations as a likely cause of increased FWHM and the results will be discussed below.

Furthermore, the relative 2Θ-shift values (Δ2Θ) of the (0110) rocking curves were determined. A color scale ranging from blue to white to red in the contour plots represents values ranging from −20 to 20 arcsec [Fig. 2 ▸(*b*)]. Most of the wafer (over 80% of the wafer area) exhibits low absolute 2Θ-shift values in between ±15 arcsec, indicating small amounts of lattice rotation around the [0001] *c* axis and therefore low prism plane twist. Generally, a spatial correlation of areas with a higher 2Θ shift to areas of higher FWHM values in Fig. 2 ▸(*a*) is visible. It is also noted that within the area marked by a black circle an increase in 2Θ shift from 8 to 18 arcsec can be measured from the adjacent area towards the center of the black circle. Additionally, a gradual increase of Δ2Θ seems to appear along the *x* position of the wafer, possibly indicating a slight curvature of the whole wafer. As suggested in the literature (Yao *et al.*, 2019 ▸, 2020 ▸), AlN wafers may exhibit a curvature of the [0004] planes due to the strain caused by the thermal gradients during growth, as well as the anisotropic cooling post-growth. The resulting convex warp of the N-face basal plane may explain the Δ2Θ of the prism planes.

**Figure 4:**
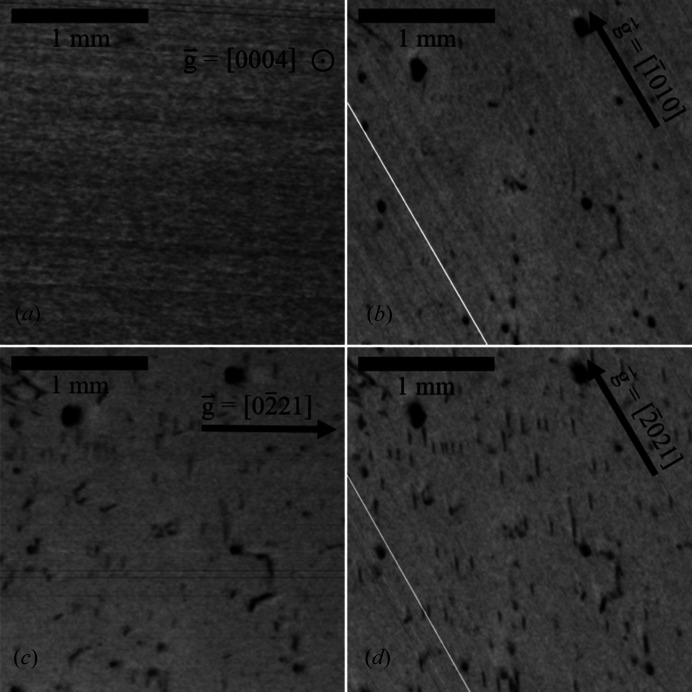
XRT transmission measurements of A3 with (*a*) 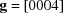, (*b*) 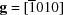, (*c*) 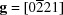 and (*d*) 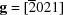. Dark spots in (*b*), (*c*) and (*d*) are due to residual damage from surface preparation steps. The diagonal white lines in images (*b*) and (*d*) are due to image stitching, while the thin horizontal lines in image (*c*) are artifacts.

**Figure 5:**
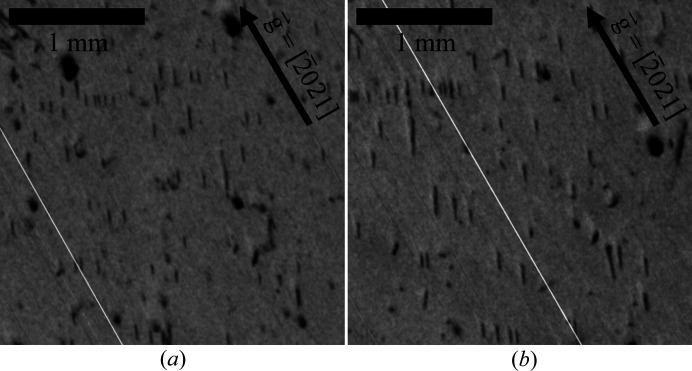
XRT 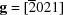 measurements of the center region of wafers (*a*) A3 and (*b*) A4. The images shown are sections of the larger areas used for DD determination. The diagonal white lines are due to image stitching.

An XRT transmission image of the same wafer A3 is depicted in Fig. 3 ▸. With 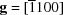 the measurement is sensitive especially to BPDs and threading edge dislocations (TEDs). Some visible contrast could be attributed to saw marks and residual damage from surface preparation steps (*e.g.* area C). Nevertheless, a detailed analysis confirms that large areas of the wafer seem to be of high quality, correlating to a low number of dislocations, which are individually discernible at higher magnification (Fig. 4 ▸ and Fig. 5 ▸).

Fig. 4 ▸ shows XRT measurements of region D in Fig. 3 ▸, which exhibits a low FWHM and 2Θ shift and is void of macroscopic defects. Furthermore, 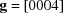 reflection topography measurements [Fig. 4 ▸(*a*)] in this region show no contrast, indicating largely the absence of threading screw dislocations (TSDs), the density of which is in detail at least two orders of magnitude lower than the total dislocation density. In contrast, 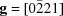 and 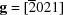 transmission measurements as seen in Fig. 4 ▸(*c*) and Fig. 4 ▸(*d*), respectively, show a high quantity of dislocations with a projected (to the basal plane) line length of less than 150 µm. With a wafer thickness of 410 µm (after surface preparation steps and etching), the inclination angle of the dislocation lines to the *c* axis is therefore less than 20°. From these geometric considerations it follows that the dislocations are most likely of a threading type.

Applying the 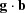 criteria reveals that short dislocation lines visible at 30° to the horizontal level in Fig. 4 ▸(*c*) are characterized by a Burgers vector of 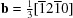, whereas the short and nearly vertical lines visible in Fig. 4 ▸(*d*) relate to a Burgers vector of 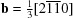. These vertical-running dislocation lines are also slightly visible in images taken with a diffraction vector of 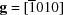 [Fig. 4 ▸(*b*)], but of reduced intensity since the diffraction intensity of 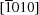 is significantly lower compared with that of 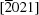 and with the very dark spots related to saw marks of former surface preparation steps, whereas the dislocation lines visible in Fig. 4 ▸(*c*) are clearly absent in Fig. 4 ▸(*b*). In contrast, these vertical lines are weakly visible in Fig. 4 ▸(*c*), which can be explained in this special case because the diffraction vector is parallel to 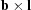, for which edge dislocations are known to show some weak contrast even though 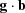 is equal to zero. However, since the projected dislocation lines run parallel to the Burgers vectors, this may point to a small screw component of these TEDs, and due to the complex diffraction contrast a low fraction of threading mixed dislocations (TMDs) of *a* + *c* type cannot be excluded, these being frequently observed in wurtzitic structures (Nakamura *et al.*, 2007 ▸; Yao *et al.*, 2020 ▸). In addition, it is remarkable that the third, symmetrically equivalent Burgers vector of 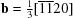 is not observed in this specific region, which is potentially connected to the fact that this group of dislocations is related to a strain field induced by an outwards-pointing thermal gradient, hence affecting only two of three potential Burgers vectors. The dislocation density determined by XRT image analysis is approximately 2 × 10^3^ cm^−2^ in the investigated region.

Image sections of 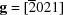 XRT transmission measurements of wafers A3 and A4 are shown in Figs. 5 ▸(*a*) and 5 ▸(*b*), respectively. These sections were selected roughly at the same lateral position within the crystal and thus trace the axial development of the dislocation density and distribution within the major growth direction along the *c* axis. Dislocation lines are not only found at the same position but also have similarly oriented 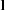 vectors.

The determination of the dislocation densities (DDs) for the two wafers resulted in the same value of 2 × 10^3^ cm^−2^ of threading-type dislocations for both wafers. As the distance between the wafers is small, the DDs are comparably low and the dislocation line vectors 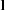 follow the growth direction; a significant reduction of the DD via annihilation becomes rather unlikely.

Some selected regions, however, show quite high dislocation densities of long parallel-running dislocation lines. Examples are those surrounding misoriented grains (Fig. 3 ▸, area A). No diffraction contrast is visible for these grains due to the high misorientation.

The position of most misoriented grains, especially visible close to the wafer edge, could be directly linked to local stress areas caused by a specific, non-optimized seed mounting. As the main focus of this paper is the investigation of the impact of different growth modes on defect formation in the more homogeneous low-defect areas, dominating most of the crystal volume, this issue will be discussed elsewhere.

Nevertheless, one circled defect cluster, not directly affected by seed mounting (Fig. 3 ▸, area B) and already highlighted in Fig. 2 ▸ as an area of increased FWHM and Δ2Θ values, was chosen for a more detailed investigation.

**Figure 6:**
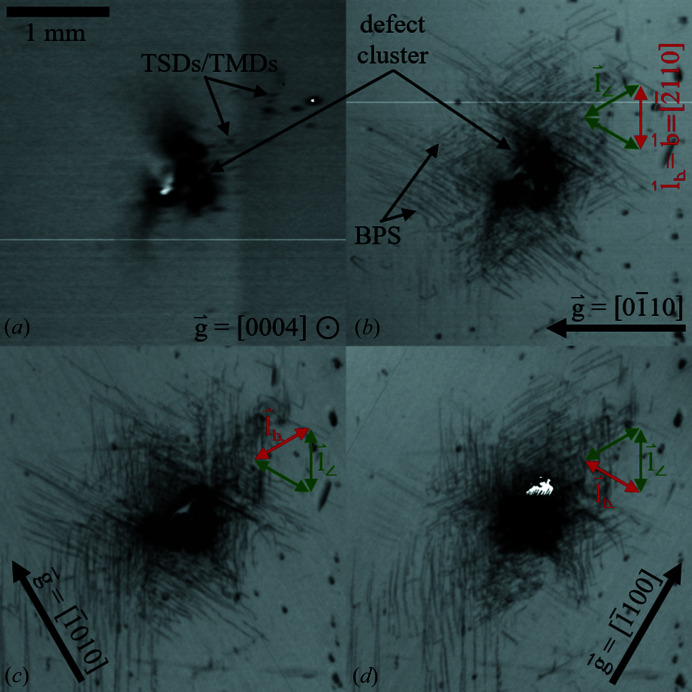
XRT measurements of a defect cluster of wafer A3: (*a*) 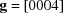, (*b*) 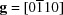, (*c*) 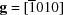 and (*d*) 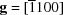. BPSs with 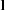 vectors of type 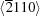 are visible. They show high contrast if their 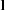 vector is oblique towards 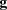 (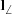 parallel to green arrows), and insignificant contrast if their 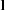 vector is perpendicular to 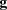 (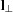) (parallel to red arrows).

Multiple XRT measurements of this area are shown at higher magnifications in Fig. 6 ▸. While Fig. 6 ▸(*a*) is a 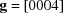 reflection measurement, Figs. 6 ▸(*b*)–6(*d*) are transmission measurements of type 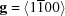 with the specific diffraction vectors 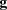 rotated by relative steps of 60° within the basal plane.

Both the reflection measurement with 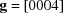 in Fig. 6 ▸(*a*) and the transmission measurement with 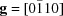 in Fig. 6 ▸(*b*) show a dark center region with a size of ∼1 mm, indicating a high density of defects affecting the basal and prism planes. Around this defect cluster, the reflection measurement shows individual, small, dark spots, indicating either TSDs or TMDs.

Further, dislocation lines are visible around the defect cluster in the transmission measurement in Fig. 6 ▸(*b*). They are running straight along the three 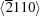 directions, forming a ‘Star of David’ pattern around the defect cluster. Due to the high projected line lengths of up to 1 mm and more, the inclination angle of the dislocation lines to the *c* axis seems to be small. Thus, the dislocations are most likely BPDs. While the Burgers vector of BPDs connecting threading dislocations has been identified as being of type 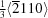, these seem to appear arc shaped or as half loops instead (Yao *et al.*, 2019 ▸). BPDs looking similar to those shown in Fig. 6 ▸(*b*) have been found adjacent to low-angle grain boundaries near wafer edges (Dudley *et al.*, 2013 ▸). However, the exact type of these dislocations has not been determined so far.

The fact that the dislocations are not visible in Fig. 6 ▸(*a*) indicates that the 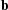 vectors of those dislocations are probably oriented in the basal plane. Comparing Fig. 6 ▸(*b*) with Fig. 6 ▸(*c*) reveals that dislocations with 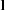 vectors oblique towards the diffraction vector 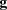 show high contrast (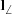), while those with 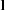 vectors perpendicular to 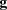 (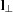) are hardly visible. Hence, in Fig. 6 ▸(*b*) with 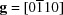, vertical dislocations of type 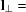 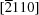 are hardly visible. With 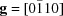 it follows from the 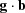 criteria that 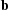 must be of type 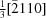 to explain the reduced contrast. As 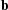 and 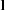 are both of the same orientation, the dark lines in Figs. 6 ▸(*b*)–6(*d*) must be basal plane screw dislocations (BPSs) of type 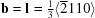.

To our knowledge, this specific dislocation type has been distinctively identified only once and very recently in PVT-grown AlN material (Yao *et al.*, 2019 ▸). The research group suggests thermal stress as the likely cause, indicated by the increased density of the BPSs near the edge of the wafer. It is also of interest to note that a similar star-shaped BPS pattern can be observed by indentation of low-dislocation-density GaN (Albrecht *et al.*, 2002 ▸). Thus, our findings support the hypothesis that the local stress field around defect clusters can be accommodated by BPSs during AlN PVT growth or transient steps like the system cool-down.

**Figure 7:**
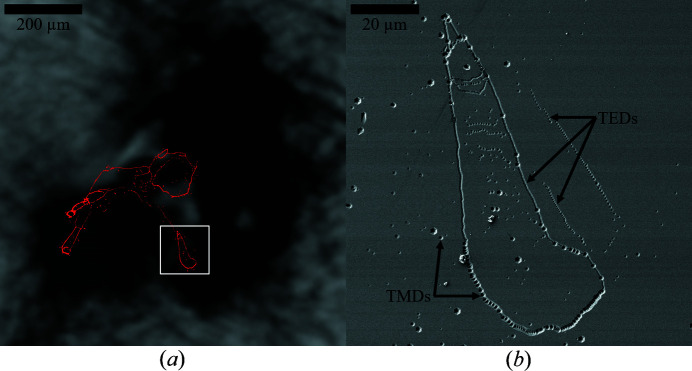
(*a*) Optical microscopy of a KOH–NaOH-etched grain boundary (red colored) stacked on top of a 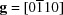 XRT reflection image from Fig. 6 ▸(*b*). A selected area of the etched grain boundary marked by the white square is shown in (*b*) as a high-resolution SEM image. Etch pits related to TEDs and TMDs are marked by black arrows.

KOH–NaOH etching of the Al side reveals distinctive lines made up of a huge number of etch pits, indicating the presence of grain boundaries. By overlaying a microscopy image montage of the whole wafer on top of the 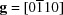 XRT image, as demonstrated in Fig. 7 ▸(*a*), it becomes apparent that the position of these grain boundaries matches the position of the dislocation clusters. The misoriented region therefore seems to be in the center of the surrounding BPSs. SEM images show that the grain boundaries consist of mostly small (∼0.8 µm) as well as some medium-sized (∼1.5 µm) etch pits, associated with TEDs and TMDs, respectively (Weyher, 2006 ▸). While TEDs compensate for angle differences from lattice plane twist, TMDs compensate also for lattice tilt. These results further confirm the X-ray measurements in which both lattice plane twist and tilt were associated with defect clusters.

Additionally, the development of these defect clusters over the height of the crystal was analyzed. Therefore, the area of the dark center region [see Fig. 6 ▸(*b*)] of six different macroscopic defect clusters visible in both wafers A3 and A4 was determined. The size varied greatly between the different clusters and no clear correlation could be extracted with regard to a size change over individual clusters between A3 and A4. However, the total defect area, defined as the sum of the visible dark center regions of the defect clusters (not considering the diffuse contrast of surrounding BPDs) within one wafer, increased by around 40% from 0.95 mm^2^ (A3) to 1.35 mm^2^ (A4).

**Figure 8:**
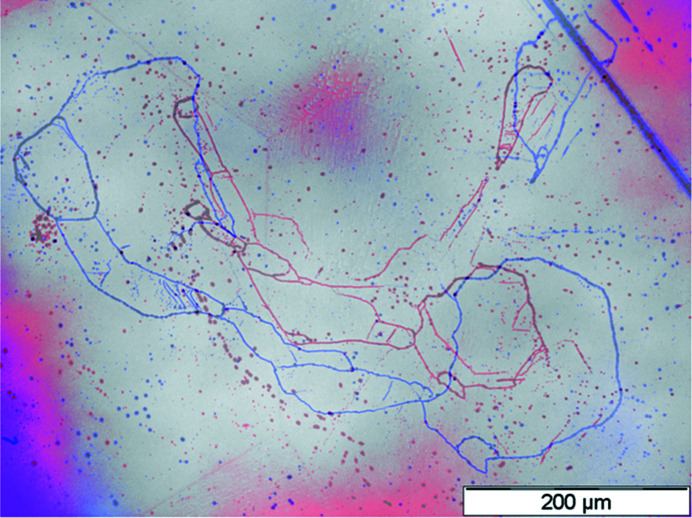
Microscopy images of the KOH–NaOH-etched Al sides of A3 and A4 in the region of defect clusters stacked on top of each other. Grain boundaries of A3 (colored red) and A4 (colored blue) can be observed.

This observation was further confirmed by comparing the etched grain boundaries in these regions. Fig. 8 ▸ shows the overlay of the etched small-angle grain boundaries of A3 (as a red line) and A4 (as a blue line). The grain boundaries of wafers A3 and A4 share a similar shape. However, there is an expansion of the enclosed area inside the boundary line from A3 to A4. According to these findings, the formation of such defect clusters needs to be inhibited, as it is not clear they can be dissolved again by a targeted strategy (*e.g.* under different growth conditions).

**Figure 9:**
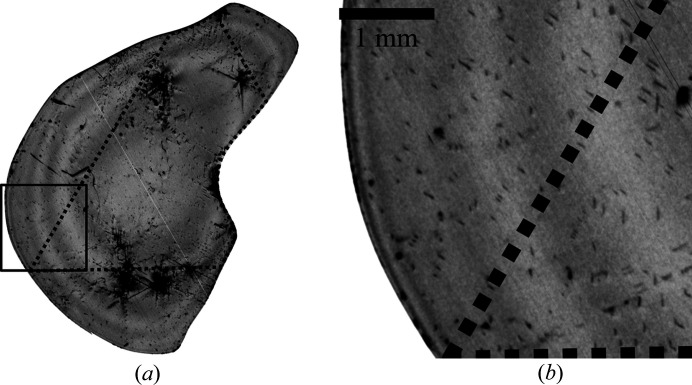
(*a*) XRT 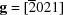 measurements of wafer B4 in transmission. The dotted triangle illustrates the border between material grown axially on top of the seed and the lateral expansion area. (*b*) Magnified section of the XRT image to compare the DDs of the two regions.

Finally, the change of the overall crystal quality in lateral *a* and *m* directions was examined. Crystal B was grown from a triangular-shaped seed [Fig. 1 ▸(*c*)] in order to facilitate enhanced lateral growth and a significant lateral expansion within a single growth run. The crystal shown in Fig. 1 ▸(*b*) was grown approximately to 4 mm thick in the *c* direction and still exhibits large 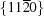 facets. The amount of lateral *a*-face growth (5–6 mm) was remarkably high and even larger than the axial growth in the *c* direction. As wafer B4 [Fig. 1 ▸(*d*)] broke during surface preparation steps, the XRT transmission measurement depicted in Fig. 9 ▸(*a*) was performed on a fragment of wafer B4. A dotted line outlines the original triangular seed and therefore the border between two different growth zones. Inside the dotted line, material could grow on top of the seed as a pure *c*-direction growth. Outside the dotted line, a significant portion of the crystal growth occurs via lateral growth.

Fig. 9 ▸(*b*) shows a magnified section of the wafer B4 XRT image. No visible differences in crystal quality could be seen along the boundary between the two growth regimes. While dislocations appear in both regimes, no significant difference in DDs with values of 1–2 × 10^3^ cm^−2^ was found.

## 4. Summary   

PVT-grown AlN samples were analyzed by high-energy X-ray diffraction and X-ray topography. In accordance with the low thermal gradient conditions employed for the growth, the measurements proved the high quality of the crystals with a low threading dislocation density of 2 × 10^3^ cm^−2^ of mainly TEDs.

The comparison of XRT images of subsequent wafers sliced from the same crystal resulted in an identical threading dislocation density, confirming that no deterioration of high-quality regions occurs during axial growth under low thermal gradient conditions.

On the other hand, locally increased FWHM and 2Θ shift could be found for misoriented grains and defect clusters. It was shown that the latter were accommodated by both grain boundaries and the formation of BPSs in adjacent areas during growth. Furthermore, the size of the defect clusters actually increased during axial growth, which indicates that the new formation of such macroscopic defects (*e.g.* due to process fluctuations or contamination of the growth front by foreign particles) needs to be inhibited, and seed selection within an AlN wafer production process must be performed very carefully to exclude the employment of seeds having defects of such type even prior to the growth process.

Most significantly, the crystal quality was maintained in expansion areas compared with the center of the crystal, even for the case when significant crystal material was added laterally within a single growth run (*i.e.* ratio of lateral to axial growth >1). This was tested and achieved by specific triangular seed shaping, enhancing the lateral growth (primarily along the *a* direction). To conclude, it was confirmed, using various X-ray techniques, that fundamentally the high quality of AlN material can be preserved within both the axial and lateral growth directions under low temperature gradient conditions and in agreement with results previously reported in the literature. Under the investigated near-equilibrium growth conditions and even with a significant diameter expansion via *a*-face growth, no kinetic factors limiting the crystal quality are apparent.
